# Formula, No. 2

**Published:** 1871-08

**Authors:** H. W. Richardson

**Affiliations:** Chicago, (formerly of Woodstock, Ill.)


ARTICLE VI.—"*Formula, No. 2.*" By H. W. RICHARDSON, M.D., Chicago, (formerly of Woodstock, Ill.)

I. S. Hawley, M.D., of Brooklyn, N. Y., in an article on "Pepsin and its Incompatibles," page 212, April No. of the Journal, takes occasion to state what no one will deny, that failures sometimes occur in administration of true and active medicinal pepsins. After enumerating some reasons for these failures, given by Chambers, he proceeds to illustrate other reasons by reference to formula written by me for the March No. of the Journal. He claims, I judge, as a matter of theory, without having tried it, that this formula fails, because the outgrowth of careless pharmacy, or utter disregard of the laws of Physiological Chemistry.

Without any disposition to occupy the space of the Journal or the time of its readers in an elaborate answer to Dr. H., we must say that we are amused at his conclusions—

1st. That "Whatever good this mixture may have done, is *not* attributable to its peptic action."

2d. That "Simple pepsin is capable of accomplishing *all* the indications here set forth."

If the Dr. can reconcile these two propositions with each other, we shall readily accord to him the honor of being ahead.

We admit freely, after submitting these matters to a practical test, that there may be conditions of the stomach requiring acids with pepsin, and other conditions requiring alkalies with pepsin, and others again where pepsin alone is *the* thing. We could give, however, were it necessary, numerous instances of marked benefit, and some of almost miraculous relief and cure, from the exhibition of the exact formula given. And we are not making it too emphatic when we say we *know* that the alkali in the formula given does not destroy or neutralize the action of the pepsin. Please understand we are no sticklers for any one formula or set of formulas, and as a rule think that every man should formulate his prescriptions to suit the exigencies of each individual case.

Our reason for using the formulas in question has grown out of a recognition of, and firm belief in, the doctrine, that the condition met with as a rule in diarrhœa, is one of *inanition;* that unfortunate babies, starved by hand-feeding or by the imperfect condition of the mother's milk, attacked by diarrhœa, are not cured or benefited, but are slain by the thousands by the routine adherents of calomel, castor oil, rhei, etc.

We admit these agents may be necessary in some rare instances, but while, perhaps, they may make the stools less disagreeable for a time by throwing into the bowels a quantity of bile which prevents their decomposition, this is done at the expense of the poor child's body, and can but contribute to the debility, and hasten the destruction of the child. If we read the laws of physiology and pathology correctly, diarrhœa is that departure from a physiological condition which involves defective vitality of mucous membranes; defective absorbing power; suspension, either partial or complete, of the gastric function; possible congestion of the portal system; irritation of the stomach and intestines; and augmented exudation of fluids. Chambers teaches that not only these departures from health, but chorea, epilepsy, teething fits, convulsive apoplexy, delirium tremens, hysteria, and some forms of mania, are familiar instances of *diseased inanition*, and that the treatment should consist in the *replacing lost nervous power*, not of restraining its excess.

It seems to us then that cathartics should be used in diarrhœa only when the prima via needs unloading; that our aim should be to *control* diseased action—either by the use of formula No. 1 or its equivalent—and restore—either by formulas Nos. 2 and 3 or their equivalents—nutrition and health.

